# Stress-responsive hydroxycinnamate glycosyltransferase modulates phenylpropanoid metabolism in *Populus*

**DOI:** 10.1093/jxb/eru192

**Published:** 2014-05-06

**Authors:** Benjamin A. Babst, Han-Yi Chen, Hong-Qiang Wang, Raja S. Payyavula, Tina P. Thomas, Scott A. Harding, Chung-Jui Tsai

**Affiliations:** ^1^Warnell School of Forestry and Natural Resources, University of Georgia, Athens, GA 30602, USA; ^2^Department of Genetics, University of Georgia, Athens, GA 30602, USA; ^3^Complex Carbohydrate Research Center, University of Georgia, Athens, GA 30602, USA

**Keywords:** Glycosylation, hydroxycinnamate glucose ester, metabolite profiling, phenylpropanoid, *Populus*, stress, UGT84A.

## Abstract

The diversity of phenylpropanoids offers a rich inventory of bioactive chemicals that can be exploited for plant improvement and human health. Recent evidence suggests that glycosylation may play a role in the partitioning of phenylpropanoid precursors for a variety of downstream uses. This work reports the functional characterization of a stress-responsive glycosyltransferase, GT1-316 in *Populus*. GT1-316 belongs to the UGT84A subfamily of plant glycosyltransferase family 1 and is designated UGT84A17. Recombinant protein analysis showed that UGT84A17 is a hydroxycinnamate glycosyltransferase and able to accept a range of unsubstituted and substituted cinnamic and benzoic acids as substrates *in vitro*. Overexpression of GT1-316 in transgenic *Populus* led to plant-wide increases of hydroxycinnamoyl-glucose esters, which were further elevated under N-limiting conditions. Levels of the two most abundant flavonoid glycosides, rutin and kaempferol-3-*O*-rutinoside, decreased, while levels of other less abundant flavonoid and phenylpropanoid conjugates increased in leaves of the GT1-316-overexpressing plants. Transcript levels of representative phenylpropanoid pathway genes were unchanged in transgenic plants, supporting a glycosylation-mediated redirection of phenylpropanoid carbon flow as opposed to enhanced phenylpropanoid pathway flux. The metabolic response of N-replete transgenic plants overlapped with that of N-stressed wild types, as the majority of phenylpropanoid derivatives significantly affected by *GT1-316* overexpression were also significantly changed by N stress in the wild types. These results suggest that UGT84A17 plays an important role in phenylpropanoid metabolism by modulating biosynthesis of hydroxycinnamoyl-glucose esters and their derivatives in response to developmental and environmental cues.

## Summary

UGT84A produces a dynamic pool of hydroxycinnamoyl-glucose esters in vegetative tissues and can modulate phenylpropanoid metabolism in response to developmental and environmental cues, such as nitrogen limitation, in *Populus.*

## Introduction

Phenylpropanoids play important roles in plant structural integrity (e.g. lignin) and defence against biotic and abiotic stressors (e.g. flavonoids, condensed tannins (CTs), and phenolic glycosides (PGs)). Their composition and abundance thus have significant impact on biomass utilization for pulp, biofuels, forage, or atmospheric carbon sequestration and on ecological interactions (Boerjan *et al.*, 2003; Dixon, 2005; Tsai *et al.*, 2006). Phenylpropanoids also possess nutritive and pharmaceutical value that can be exploited for human health applications (Kammerer *et al.*, 2005; Ververidis *et al.*, 2007).

Hydroxycinnamates derived from phenylalanine are the simplest of the phenylpropanoids and are precursors to other more elaborate phenylpropanoid metabolites, such as lignin and flavonoids. Hydroxycinnamates accumulate in a great variety of ester or amide conjugates with monosaccharides, organic acids, lipids, and amines (Strack, 2001). These hydroxycinnamate conjugates have been implicated in pathogen response (Bernards *et al.*, 1991; Beimen *et al.*, 1992), symbiont interactions (Weiss *et al.*, 1999), and ultraviolet (UV) protection (Landry *et al.*, 1995; Sheahan, 1996; Grace *et al.*, 1998; Kolb *et al.*, 2001). Activated hydroxycinnamates in the forms of CoA or glucose esters are major acyl donors for modification of secondary metabolites (Teusch *et al.*, 1987; Gläßgen and Seitz, 1992; Mock and Strack, 1993) into end products with altered physicochemical properties and hence bioactivities (D’Auria, 2006; Yoshida *et al.*, 2009). Hydroxycinnamoyl esters also cross-link with lignocellulosic polymers, thereby affecting cell-wall strength and biomass utilization (Ralph *et al.*, 2004). Consistent with their multiple roles in phenylpropanoid metabolism, genetic perturbations affecting hydroxycinnamates or hydroxycinnamate conjugates have wide-ranging effects on phenylpropanoid carbon allocation between different branch pathways (Sinlapadech *et al.*, 2007; Lanot *et al.*, 2008; Clauß *et al.*, 2011; Mittasch *et al.*, 2013).

Hydroxycinnamate glucose esters represent the most common form of hydroxycinnamate conjugates in plants (Corner and Swain, 1965). Their synthesis depends on family 1 glycosyltransferases (GT1), which catalyse the transfer of sugars to small acceptor molecules (Bowles *et al.*, 2006). To date, only a handful of GT1s have been shown to catalyse the formation of hydroxycinnamoyl-glucose esters, and all belong to the UGT84A subfamily of group L of plant GT1 proteins (Milkowski *et al.*, 2000a; Lim *et al.*, 2001; Lunkenbein *et al.*, 2006; Mittasch *et al.*, 2007). *Arabidopsis* UGT84A2 and its *Brassica napus* (oilseed rape) orthologue UGT84A9 represent the best characterized members. Both enzymes exhibit a specific substrate preference for sinapic acid and produce sinapoyl-glucose as the acyl donor for the biosynthesis of sinapoyl-malate and sinapoyl-choline, the major soluble phenylpropanoids in Brassicaceae (Milkowski *et al.*, 2000a; Lim *et al.*, 2001). Whereas sinapoyl-malate functions as a UV protectant in leaves (Landry *et al.*, 1995), sinapoyl-choline accumulates at high levels in seed of these species (Hüsken *et al.*, 2005). In strawberry (*Fragaria × ananassa*), the fruit-specific UGT84A6 exhibited a slight substrate preference for cinnamic acid, and the most significant effect of its antisense downregulation was reduced levels of the flavour constituent cinnamoyl-glucose (Lunkenbein *et al.*, 2006). All other characterized UGT84A proteins, such as *Arabidopsis* UGT84A1, A3, and A4 (Milkowski *et al.*, 2000b; Lim *et al.*, 2001) and oilseed rape UGT84A10 (Mittasch *et al.*, 2007), utilize multiple hydroxycinnamate substrates *in vitro*, but their *in vivo* functions remain poorly understood.

This study describes the identification and characterization of UGT84A orthologues from *Populus*, a species known for its large and diverse reserves of phenylpropanoids (Tsai *et al.*, 2006). *Populus* harbours three UGT84A members—GT1-315 (UGT84A19), GT1-316a (UGT84A18), and GT1-316 (UGT84A17)—located in a tandem block with high sequence similarity. UGT84A17 exhibited stress-responsive expression and broad *in vitro* activities toward various hydroxylated and/or methoxylated cinnamic and benzoic acids. Overexpression of UGT84A17 in transgenic *Populus* led to hyperaccumulation of hydroxycinnamate glucose esters, especially caffeoyl-, 4-coumaroyl-, and cinnamoyl-glucose esters. Widespread changes in phenylpropanoids were also observed, supporting a role of UGT84A17 in modulating phenylpropanoid metabolism.

## Materials and methods

### Phylogenetic analysis

*Populus* GT1 sequences annotated by Geisler-Lee *et al.* (2006) based on the *Populus* genome v1.0 were used for initial phylogenetic analysis with *Arabidopsis* GT1 family (Li *et al.*, 2001) to identify group L orthologues. The gene models were cross-referenced with the *Populus* genome v3.0 to obtain updated gene models for phylogenetic analysis, along with other experimentally characterized group L members, using the *Arabidopsis* group E member UGT72B1 as outgroup. Sequence alignment was performed using the MAFFT program housed on the EMBL-EBI server (http://www.ebi.ac.uk/Tools/msa/). The alignment output was imported into MEGA5 (Tamura *et al.*, 2011) for phylogenetic tree reconstruction using the maximum-likelihood method and the Jones–Taylor–Thornton (JTT) substitution matrix with 500 bootstrap iterations.

### Recombinant PfaGT1-316 analysis and enzyme assays

A *Populus fremontii* × *angustifolia* expressed sequence tag (MTUNUL1.P64.D01, GenBank accession DY801582) matching the predicted GT1-316 in the *Populus trichocarpa* genome was fully sequenced and used for subcloning. The coding region (with the start codon converted to CTG) was amplified by PCR using gene-specific primers that introduced 5′-*Bam*HI and 3′-*Sma*I sites (Supplementary Table S1 available at *JXB* online) and cloned into pCRII-TOPO (Invitrogen/Life Technologies, Grand Island, NY, USA). After sequencing confirmation, the *Bam*HI and *Sma*I fragment was subcloned into pGEX-2TK (GE Healthcare, Piscataway, NJ, USA) and transformed into *Escherichia coli* BL21. Recombinant proteins were purified using a glutathione sepharose purification kit (GE Healthcare).

Activity of recombinant GT1-316 was first tested using 5mM UDP-glucose as the sugar donor and a variety of potential acceptor substrates (e.g. phenylpropanoids, terpenoids, indole acetic acid, zeatin) at 1mM. Kinetic analysis was performed using phenylpropanoid substrates ranging from 50 to 500 μM. Reaction conditions were based on Lim *et al.* (2001) and contained 0.2 μg protein, 100mM Tris (pH 7.5), 1mM DTT, 2mM EGTA, and 0.2mg ml^–1^ BSA in 50 μl. After prewarming at 30 °C for 75 s, the reaction was started by addition of UDP-glucose and terminated after 5min by snap freezing in liquid nitrogen. Control reactions were stopped immediately after adding UDP-glucose. The protein was denatured by addition of 60 μl acetonitrile with 0.2mM ^13^C_6_-cinnamic acid as internal standard. Following centrifugation, the supernatant was dried under vacuum to remove acetonitrile prior to analysis on an Agilent 1200 HPLC, equipped with a diode array detector and a 6220 accurate-mass time-of-flight (TOF) mass spectrometer (Agilent Technologies, Wilmington, DE, USA). Samples (1 μl each) were separated on a reversed-phase column (ZORBAX Eclipse Plus C18, 2.1×150mm, 3.5 µm; Agilent) for quantification of UDP released from UDP-glucose during the reaction, which allowed one quantification method for all glucose acceptor substrates. *N*,*N* dimethylhexylamine (DMHA) was included in the mobile phase as an ion-pairing agent (Cordell *et al.*, 2008). The gradient of mobile phase A (95% water, 5% acetonitrile, 5mM DMHA, pH 7) to mobile phase B (95% acetonitrile, 5% water, 5mM DMHA) was linear from 5% B to 30% B over 3min, and then linear up to 40% B over 3.5min, at a flow rate of 0.3ml min^–1^. The electrospray ionization was set in negative mode, with gas temperature 300 °C, drying gas 11 l min^–1^, nebulizer pressure 206.8 kPa (30 psig), capillary voltage 3500V, and fragmentor 125V. UDP was detected with a diode array detector at 260nm and by MS using the extracted ion chromatogram at *m/z* 402.9935 (expected *m/z* 402.9943). UDP concentration was estimated by a standard curve using authentic UDP (Sigma, St Louis, MO, USA). Glucose ester products were confirmed by MS and a mild alkaline treatment (Lim *et al.*, 2001). Briefly, the assay products were incubated with 0.1M NaOH at room temperature for 1hr, and neutralized with 3M sodium acetate (pH 5.2) prior to HPLC-MS/TOF analysis.

### Transgenic *Populus* production and N stress experiments

The coding region of PfaGT1-316 was PCR amplified using gene-specific primers (Supplementary Table S1 available at *JXB* online), cloned into pCRII-TOPO (Invitrogen), and sequence-confirmed. The insert was digested by *Spe*I and *Eco*RV and subcloned into pCambia1302 behind the CaMV 35S promoter in a sense orientation. The construct was mobilized into *Agrobacterium tumefaciens* pMP90 and transformed into *Populus tremula* × *alba* (clone INRA 717-IB4) using standard methods (Meilan and Ma, 2006). Independent transgenic lines confirmed by PCR were transplanted to soil and maintained in a glasshouse. Plants were fertilized weekly with a 15% solution of Miracle-Gro Water Soluble All Purpose Plant Food (Scotts, Marysville, OH, USA). Leaf tissues (leaf plastochron index LPI-5) from the original transformants were taken for initial screening analyses.

Selected plants were vegetatively propagated for N-stress experiments. Rooted cuttings were transferred to hydroponic culture in perlite pots, with N maintained at 2.5mM (ammonium/nitrate 4:1) as described (Harding *et al.*, 2005). Plants from wild-type (WT) and three transgenic lines were distributed evenly among eight hydroponic tubs. Nutrient solutions were replaced weekly and deionized water was added daily as necessary to maintain volume, with pH maintained at ~5.8. When plants were approximately 1 m tall, they were randomly divided into two groups that received either full (2.5mM) or reduced (0.25mM) N levels with the same ammonium/nitrate molar ratio, and the treatment lasted for 13 days. Plant heights, basal stem diameter (2cm above the perlite surface), and leaf lengths were measured at regular intervals. Leaf (LPI-2 and 7), young stem (internodes between LPI-0 and LPI-4), phloem (bark) and xylem (de-barked stem) from internodes between LPI-7 and LPI-12, and coarse root tissues were harvested, snap frozen, and ground to a fine powder under liquid nitrogen. An aliquot was freeze dried for metabolic analysis and the rest stored at –80 °C until use.

### Quantitative real-time PCR

Various tissues from *Populus tremuloides* (clone 271) and *P. tremula × alba* (clone 717-1B4) as described in Payyavula *et al.* (2011) or from the N stress experiments were used for RNA extraction by the CTAB method (Chang *et al.*, 1993). DNase treatment, cDNA synthesis, and quantitative real-time PCR (qRT-PCR) were conducted as described (Tsai *et al.*, 2006), using the ABsolute qRT-PCR SYBR Green Mix (ABgene/Thermo Fisher Scientific, Pittsburgh, PA, USA) and a Mx3005P Real-Time PCR system (Stratagene, La Jolla, CA, USA). Relative transcript abundance was estimated by the ΔCT method (Tsai *et al.*, 2006), using the geometric mean of three stable housekeeping genes (elongation factor 1-β, α-tubulin 4, and ubiquitin-conjugating enzyme E2) for normalization (see Supplementary Table S1, available at *JXB* online, for gene-specific primers).

### Metabolite extraction and HPLC-MS/TOF analysis

Freeze-dried tissues (10mg) were extracted in ice-cold methanol in an ultrasonicator bath for 5min. The extracts were clarified by centrifugation and stored at –80°C or analysed directly (1 μl) by HPLC-MS/TOF for nontargeted profiling as described in Xue *et al.* (2013). Metabolite data were processed by MassHunter Qualitative Analysis software (Agilent) using the ‘Molecular Feature Extraction’ function. After removing noise (signal-to-noise <150 and absolute ion counts <500), the software groups chemically related ions (isotopes, formide adducts, and dimers) to identify putative compounds (ion groups) in negative mode and generate molecular formulas (mass error≤10 ppm). The resultant files were aligned using Mass Profiler (Agilent) to generate a list of target compounds, which was filtered to retain those that were present in at least half of the samples from either WT or the transgenic group. The list was then converted to XML format using a custom script and imported into the MassHunter Quantitative Analysis software (Agilent) for peak fitting and integration to obtain abundance values. Internal standard (^13^C_6_-cinnamic acid)-normalized and tissue mass-corrected abundance values were used for statistical analysis. Separate comparisons for genotypic effects within each N regime, and N effects within genotype were made for each tissue using SLIM in R (Wang *et al.*, 2011), with statistical significance determined by either a *P*-value or a *Q*-value cut off of 0.05.

For metabolites that were significantly affected by the transgene expression, their *m/z* and tentative molecular formulas obtained from the MassHunter Qualitative Analysis software were used to search the mass spectral databases KNApSAcK (Afendi *et al.*, 2012), Metabolome Tomato Database (MoToDB, Moco *et al.*, 2006), and Dictionary of Natural Products (http://dnp.chemnetbase.com) to assign putative identity. Where possible, compound identities were confirmed by authentic standards (Supplementary Table S3), including the phenolic glucose esters obtained from *in vitro* GT1-316 assays.

### GC-MS analysis of wall-bound phenolics

An aliquot (20mg) of freeze-dried LPI-7 was extracted three times in 1ml methanol/chloroform (33:67, v/v), followed by 100% methanol and then water, 15min each by sonication at room temperature. The pellet was resuspended in 1ml 2M NaOH with methoxybenzoic acid as internal standard, and incubated on an orbital shaker (800rpm) overnight at room temperature. After centrifugation, the supernatant was adjusted to pH ~5 using 8M HCl and extracted three times with 500 µl water-saturated ethyl acetate. The pooled ethyl acetate fractions were evaporated to dryness and resuspended into 200 µl acetonitrile. A portion (150 µl) of the sample was used for derivitization and GC-MS analysis following conditions detailed in Xue *et al.* (2013). The identity of hydroxycinnamic acids was confirmed by authentic standards.

### Lignin and CT analyses

Lignin content and syringyl-to-guaiacyl monolignol (S/G) ratio were determined by pyrolysis molecular beam mass spectrometry at the Complex Carbohydrate Research Center according to Sykes *et al.* (2009), using freeze-dried stem xylem samples. Condensed tannins (CTs) were analysed by a modified n-butanol-HCl method (Porter *et al.*, 1986), using purified CTs from *Populus* leaves as standards according to Harding *et al.* (2005).

### Statistics

Unless otherwise noted, one-way or two-way ANOVA was performed for comparisons between treatments and/or genotypes using SigmaPlot v.12.3 (Systat Software, San Jose, CA, USA). The Tukey multiple comparison correction was used where appropriate. Gene expression data from qRT-PCR were log transformed prior to statistical comparison to approximate a normal distribution, as indicated by the Shapiro–Wilk test.

## Results

### Identification of a stress-responsive GT1-315/316 gene cluster

**Fig. 1.**
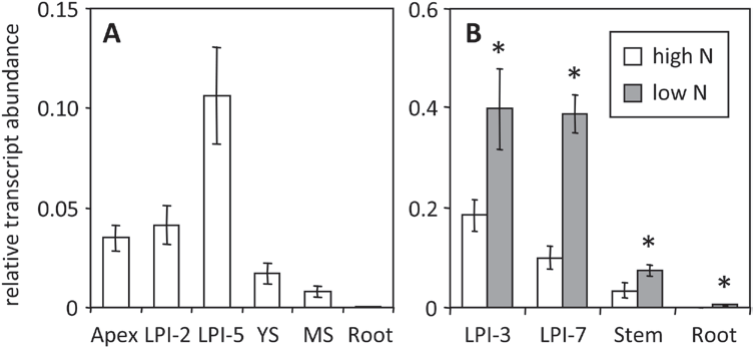
Relative transcript abundance of *Populus GT1-315/316* gene cluster in different tissues. (A) *Populus tremula* × *alba* clone 717-1B4. (B) *P. tremuloides* clone 271 grown under N-replete (high N) or N-limited (low N) conditions. Asterisks indicate statistically significant N treatment effects.

Two Affymetrix probe-sets (PtpAffx.125962.1.S1_at and PtpAffx.18005.2.A1_a_at) corresponding to previously annotated *Populus* GT1-315 and GT1-316 (Geisler-Lee *et al.*, 2006) were found to exhibit elevated expression in response to multiple stress treatments (e.g. N limitation, wounding, and detopping) and in multiple genotypes (Supplementary Fig. S1 available at *JXB* online; Yuan *et al.*, 2009; Tuominen *et al.*, 2011). In the current (v3.0) genome release, these probes match three gene models—Potri.009G095100 (GT1-316), Potri.009G095300 (GT1-316a, not previously annotated), and Potri.009G095400 (GT1-315)—that share a high degree of sequence identity (hereafter referred to as the GT1-315/316 cluster). Thus, the stress-responsive expression that the current study observed likely reflected the collective response of this gene cluster. To verify their expression, qRT-PCR was conducted in two separate experiments, using primers designed to amplify all members of the GT1-315/316 cluster. In *P. tremula* × *alba* clone 717-1B4, transcript levels of *GT1-315/316* were highest in leaves, lower in stems, and very low in roots (Fig. 1A). A similar tissue expression pattern was found in *P. tremuloides* clone 271. Consistent with the microarray findings, transcript levels of *GT1-315/316* were elevated (two-way ANOVA *P*_N-treatment_<0.001, *P*_tissue_<0.001) in *P. tremuloides* plants that were grown under N-limiting conditions (Fig. 1B).

### Phylogenetic analysis

**Fig. 2.**
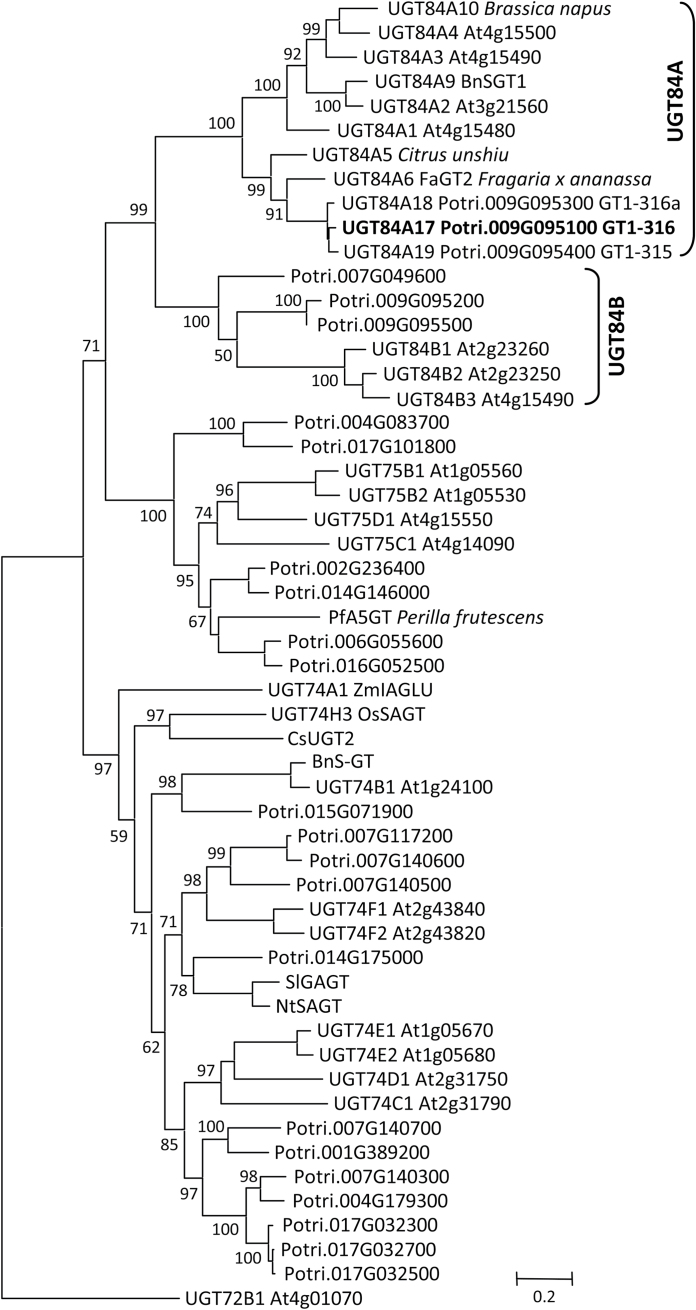
Maximum-likelihood tree of group L glycosyltransferases from *Populus* and *Arabidopsis*, along with experimentally characterized members from other species. An *Arabidopsis* group E member (UGT72B1) was used as outgroup. Bootstrap support for the branches is shown. *Populus* gene models were from the genome release v3.0 (Phytozome), whereas the *Arabidopsis* sequences were from TAIR10. GenBank accession numbers for the other sequences are: *Brassica napus* UGT84A9/BnSGT1 (AF87143), UGT84A10 (CAJ77650), and BnS-GT (AAL09350); *Crocus sativus* CsUGT2 (Q6X1C0); *Fragaria × ananassa* FaGT2/UGT84A6 (Q66PF4); *Citrus unshiu* UGT84A5 (BAA93039); *Nicotiana tabacum* NtSAGT (AF190634); *Oryza sativa* UGT74H3/OsSAGT (BAD34358); *Perilla frutescens* var*. crispa* PfA5GT (BAA36421); *Solanum lycopersicum* SlGAGT (CAI62049); and *Zea mays* UGT74A1/ZmIAGLU (AAA59054). UGT code assigned by the UDP Glucuronosyltransferase Nomenclature Committee is included when available.

Phylogenetic analysis showed that the deduced GT1-315/316 proteins are most closely related to the UGT84A subfamily (Fig. 2), belonging to group L of plant GT1s (Li *et al.*, 2001). They were assigned UGT84A17 (GT1-316), UGT84A18 (GT1-315), and UGT84A19 (GT1-316a) by the UDP Glucuronosyltransferase Nomenclature Committee. The UGT84A clade included several biochemically characterized members known to catalyse the formation of hydroxycinnamate glucose esters. Examples are the sinapate-specific UGT84A9 (oilseed rape BnSGT1, Milkowski *et al.*, 2000a) and UGT84A2 (*Arabidopsis* At3g21560; Lim *et al.*, 2001), the cinnamic acid-biased UGT84A6 (FaGT2) from strawberry (Lunkenbein *et al.*, 2006), and several other members with a broad substrate specificity: UGT84A1 (At4g15480), UGT84A3 (At4g15490), and UGT84A4 (At4g15500) from *Arabidopsis* (Milkowski *et al.*, 2000b; Lim *et al.*, 2001) and UGT84A10 from oilseed rape (Mittasch *et al.*, 2007). This strongly supported UGT84A clade was sister to another strongly supported branch that includes the *Arabidopsis* indole-3-acetic acid glucosyltransferase (At2g23260) of the UGT84B subfamily (Jackson *et al.*, 2001). Taken together, the strong phylogenetic association of GT1-315/316 with UGT84A members supports their potential involvement in phenylpropanoid metabolism.

### Biochemical characterization of GT1-316

**Fig. 3.**
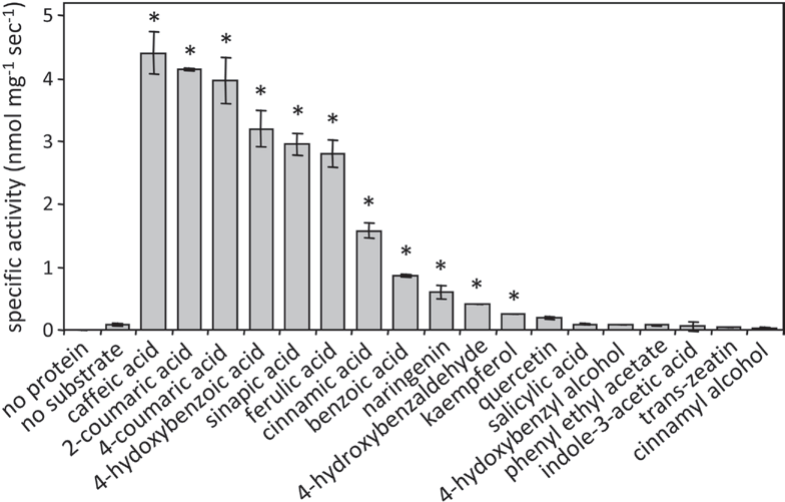
*In vitro* enzyme activity of recombinant PfaGT1-316 with various substrates. Specific activity was measured as UDP released from UDP-glucose during the glycosylation reaction. Asterisks inidicate reaction products verified by HPLC-MS/TOF (Supplementary Fig. S2 and Table S1).

Data mining of *Populus* expressed sequence tag collections (Ranjan *et al.*, 2004; unpublished data) identified one candidate full-length GT1-315/316 clone (DY801582) derived from *P. fremontii × angustifolia* (genotype NUL). This clone was fully sequenced and found to share 99% coding sequence identity with GT1-316, followed by GT1-315 and GT1-316a (~97%) of the *P. trichocarpa* genome (Tuskan *et al.*, 2006). The clone was therefore named PfaGT1-316 (GenBank accession KF552072). The coding region of PfaGT1-316 was expressed in *E. coli* for *in vitro* protein characterization. The purified recombinant PfaGT1-316 protein exhibited activity for 11 out of the 18 potential glucose acceptor substrates tested (Fig. 3, Supplementary Fig. S2, Supplementary Table S2). The PfaGT1-316 activity was higher for substituted hydroxycinnamic acids and 4-hydroxybenzoic acid than for unsubstituted cinnamic and benzoic acids. The activity with flavonoid substrates was very low or below detection. Kinetic analysis was performed for eight phenolic acids and two flavonoids (naringenin and kaempferol). PfaGT1-316 exhibited the highest catalytic activities and turnover rates toward caffeic acid, 4-coumaric acid, 4-hydroxybenzoic acid, 2-coumaric acid, ferulic acid, and sinapic acid as glucose-acceptors, while flavonoids were relatively poor substrates (Table 1). The hydroxycinnamate conjugates were hydrolysable by a mild alkaline treatment, suggesting that PfaGT1-316 preferentially catalyses the formation of glucose esters rather than *O*-glucosides (Supplementary Fig. S3), similarly to the other UGT84A orthologues (Lim *et al.*, 2001).

**Table 1.** Enzyme kinetics of recombinant PfaGT1-316 as determined by Lineweaver-Burke plotData represent mean and SD from three independent batches of protein purification, each with at least three technical replicates. ND, *K*_m_ for kaempferol could not be determined due to poor activity.

Substrate	*V*_max_ (pkat)	*K*_m_ (mM)	*k*_cat_ (s^–1^)	*k*_cat_/*K*_m_ (mM^–1^ s^–1^)
Caffeic acid	15.85±4.50	0.22±0.09	6.49±1.84	31.3±4.3
4-Coumaric acid	15.07±2.55	0.62±0.12	6.17±1.05	10.1±0.3
4-Hydroxybenzoic acid	10.61±2.95	0.72±0.33	4.35±1.21	6.6±2.0
2-Coumaric acid	8.99±3.44	0.11±0.05	3.68±1.41	35.9±3.9
Ferulic acid	7.93±1.27	0.15±0.02	3.25±0.52	22.1±1.6
Sinapic acid	7.55±2.12	0.13±0.05	3.09±0.87	24.7±3.0
Benzoic acid	3.68±0.89	1.59±0.41	1.51±0.36	1.0±0.1
Cinnamic acid	3.42±0.59	0.30±0.07	1.40±0.24	4.7±0.9
Naringenin	1.99±0.31	0.22±0.10	0.81±0.13	4.3±1.6
Kaempferol	0.55±0.13	ND	0.23±0.05	ND

### Overexpression of *PfaGT1-316* in *Populus*

To investigate the *in vivo* role of GT1-316, transgenic *P. tremula* × *alba* (717-1B4) that overexpressed *PfaGT1-316* under the CaMV 35S promoter were generated. Based on qRT-PCR screening of 16 independent transgenic lines, three (A, D, and H) with >50-fold elevated *GT1-316* transcript levels (Supplementary Fig. S4A) were selected for metabolite analysis. The expanding leaves (LPI-5) of *PfaGT1-316* transgenic plants accumulated ~15-fold higher levels of caffeoyl-glucose compared to WT (Supplementary Fig. S4B). Levels of 4-coumaroyl-glucose, feruloyl-glucose, and cinnamoyl-glucose also increased, but to a much lesser extent (1.5–3-fold) (Supplementary Fig. S4C–E).

**Fig. 4.**
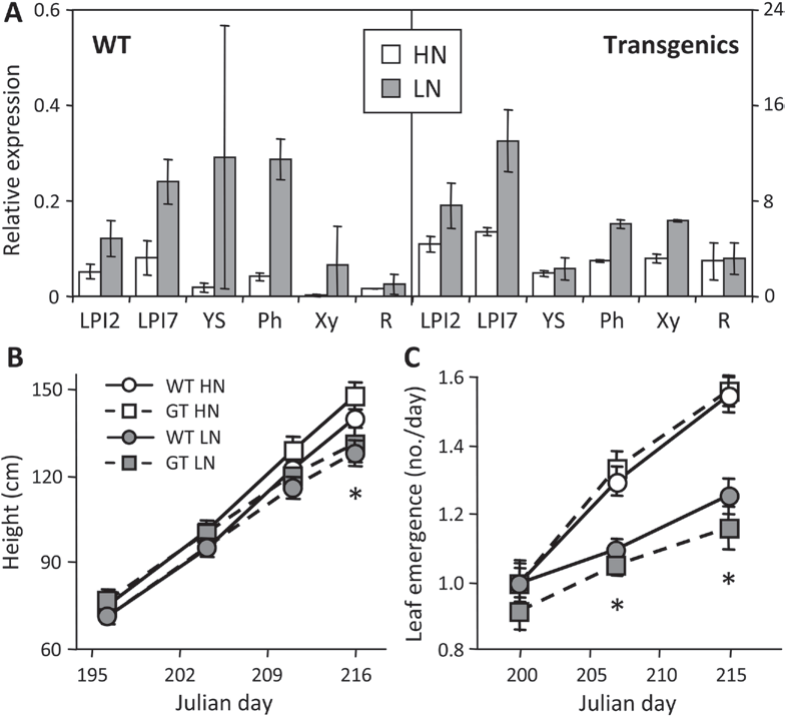
Molecular and growth characterization of transgenic *Populus*. (A) Relative transcript abundance of the *GT1-315/316* cluster in WT and transgenic plants under N-replete (HN) or N-limited (LN) treatments. Leaves (LPI2, LPI7), young stem (YS), phloem (Ph), xylem (Xy), and coarse roots (R) were analysed. Values are mean and SD of three biological replicates from line D. (B and C) Height growth (B) and leaf emergence (C) rates of WT and transgenic (GT) plants. Values are mean and SE of 15 biological replicates, pooled from lines A, D, and H. Asterisks indicate significant LN treatment effects.

WT and transgenic PfaGT1-316 plants were subjected to hydroponic N manipulation to perturb plant growth and phenylpropanoid metabolism. Low N availability clearly stressed the plants, causing leaf yellowing and reduced shoot growth and leaf emergence rates (Fig. 4; see also Supplementary Fig. S5 available at *JXB* online). Transcript levels of endogenous *GT1-315/316* were upregulated by 1.5–30-fold, depending on the tissue, in response to N stress in WT (Fig. 4A). In comparison, the magnitude of *PfaGT1-316* overexpression (relative to the endogenous *GT1-315/316*) in transgenic plants was much greater, by 67–1500-fold at high N or by 8–121-fold at low N (Fig. 4A). There was little or no morphological phenotype of *PfaGT1-316* transgenic plants regardless of N status (Fig. 4B, C). However, under N-limited conditions, 53% of the *PfaGT1-316* plants ceased growth and set buds by the end of the experiment, but only 20% of the WT plants did so (data not shown).

### Metabolic consequences of *PfaGT1-316* overexpression in *Populus*

**Fig. 5.**
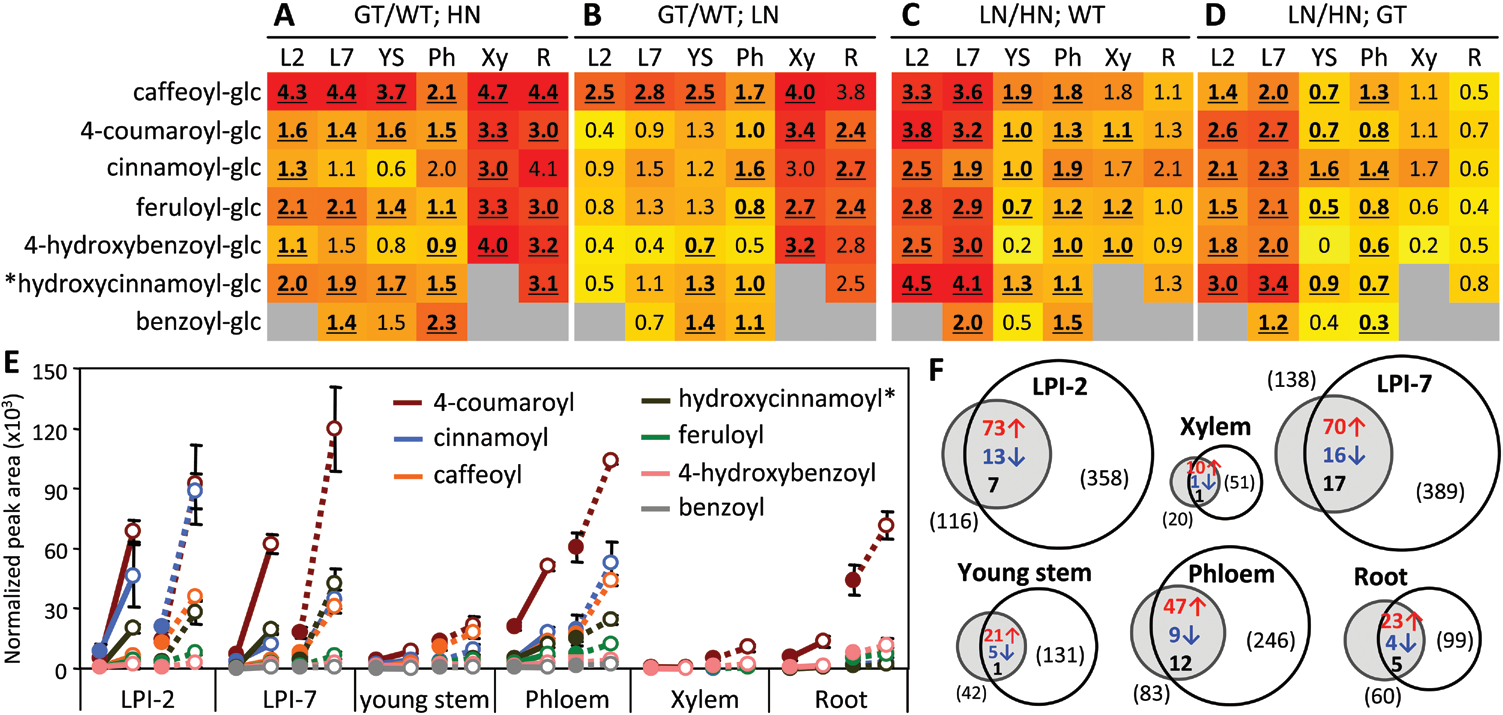
Effects of *PfaGT1-316* overexpression and N limitation on hydroxycinnamate glucose ester accumulation. (A and B) Transgenic effects (GT/WT) under high-N (A) or low-N (B) conditions. (C and D) N stress response (LN/HN) in WT (C) and transgenic (D) plants. Shown are log2-transformed ratios in heatmaps. Values in bold-face with underline denote statistical significance (*Q* <0.05). Grey indicates below detection. (E) The same data plotted by normalized peak areas to illustrate the relative abundance of the glucose esters (solid symbols, HN; open symbols, LN; solid lines, WT; dashed lines, transgenic). Values are mean and SD of three biological replicates. The asterisk indicates an unknown hydroxycinnamoyl-glucose ester with a *m/z* of 325.0921. (F) Venn diagrams showing overlap of significantly changed metabolites (*P*<0.05) due to transgenic (GT/WT under HN, grey circles) or N (LN/HN in WT, white circles) manipulation. The numbers of metabolites with increased (red) or decreased abundance (blue), or inconsistent changes (black) are shown in the overlap, with the total number of significantly changed metabolites noted in parentheses.

Several (hydroxyl)cinnamate/benzoate glucose esters were detected in the *P. tremula* × *alba* tissues examined, including six of the PfaGT1-316 glycosylation products *in vitro*: caffeoyl-, 4-coumaroyl-, 4-hydroxybenzoyl-, feruloyl-, cinnamoyl-, and benzoyl-glucose (Fig. 5). All six glucose esters were more abundant in transgenic plants than in WT across all tissues and N regimes (Fig. 5A, B). Also elevated in transgenic plants was a putative hydroxycinnamoyl-glucose with a matching *m/z* but a different retention time as compared to 2- or 4-hydroxycinnamoyl-glucose esters. In contrast, the *in vitro* PfaGT1-316 glycosylation products of naringenin and kaempferol were not detected in *Populus* tissues. The results were consistent with the observed broad *in vitro* substrate preference of PfaGT1-316 toward various cinnamic and benzoic acid derivatives, but not flavonoids.

Across all tissues and N treatments, caffeoyl-glucose exhibited the greatest fold-increase in transgenic plants (Fig. 5A, B), due partly to its relatively low abundance in WT plants (Fig. 5E, Supplementary Table S3). Levels of the more abundant 4-coumaroyl-glucose and cinnamoyl-glucose (LPI-2) also showed large absolute increases in transgenic plants, although the fold-change was lower than that of caffeoyl-glucose (Fig. 5A, B, E). Most of the hydroxycinnamoyl- and benzoyl-conjugates were present at low abundance in xylem and roots, and overexpression of *PfaGT1-316* resulted in large fold changes from near absence in WT (Fig. 5A, B, E, Supplementary Table S3 available at *JXB* online). Under N-limited conditions, accumulation of most of these glucose conjugates was stimulated, particularly in leaves, regardless of genotype (Fig. 5C–E). Some hydroxycinnamates, such as 4-coumaric, caffeic, and ferulic acids are known to accumulate in *Populus* as wall-bound phenolics (Gou *et al.*, 2008). No consistent transgenic or N treatment effects on the abundance of wall-bound phenolics were observed (Supplementary Fig. S6).

Nontargeted HPLC-MS/TOF profiling of secondary metabolites revealed additional metabolic changes due to *PfaGT1-316* overexpression or to N-limitation. In general, leaves had the most complex metabolite profiles, while xylem extracts had the lowest number of detectable metabolites. Statistical analysis showed an overall stronger effect of N-limitation than *PfaGT1-316* overexpression on *Populus* metabolism (Fig. 5F). The leaf and phloem metabolomes were affected the most by either perturbation, based on *P*≤0.05 (Fig. 5F) or *Q*≤0.05 (Supplementary Table S3). Consistent with the stress-responsive nature of *GT1-316* (Figs 1 and 4A, Supplementary Fig. S1), metabolite changes due to *GT1-316* overexpression overlapped substantially with those induced by N stress, accounting for 62–74% of significantly changed metabolites in green tissues and 45–55% in xylem and root of transgenic plants (Fig. 5F). A majority of the significantly affected metabolites showed increased abundance in PfaGT1-316 transgenic plants (Fig. 5F), and most of them were predicted to be phenylpropanoid derivatives, including conjugates of various flavonoids and di- and tri-glycosides of hydroxycinnamates (Supplementary Table S3). The latter included several putative caffeic acid derivatives (e.g. dicaffeoylquinates, caffeoyl-salicin, hydroxycinnamoyl-salicin), in addition to the hydroxycinnamoyl-glucose esters discussed above. Relatively fewer metabolites showed decreased abundance in transgenic plants, especially in leaves. Among compounds that decreased in concentration were rutin (quercetin-3-*O*-rutinoside) and kaempferol-3-*O*-rutinoside, the two most abundant flavonoids in leaves (Supplementary Table S3). Together, these results suggested that UGT84A-mediated hydroxycinnamate glycosylation plays an important role in phenylpropanoid metabolism during *Populus* stress response.

### Major phenylpropanoids were not affected in *PfaGT1-316* transgenic plants

The effects of *PfaGT1-316* overexpression on accumulation of major phenylpropanoid end products (PGs, CTs, and lignin) were examined. PGs such as salicortin and tremulacin were most abundant in leaves, while CTs and lignin were present at highest levels in roots and xylem, respectively, of clone 717-1B4 (Supplementary Fig. S7). Any transgenic effects on PGs and lignin (both content and S/G ratio) were minor and inconsistent across tissues and N status (Supplementary Fig. S7A, C, D). CTs were also largely unaffected by *PfaGT1-316* overexpression, but unlike PGs, increased significantly in response to N-limitation, except in xylem where CTs were barely detected (Supplementary Fig. S7B). Overall, the data suggested that *PfaGT1-316* overexpression had little effect on PGs, CTs, and lignin in *Populus*.

### Transcript levels of phenylpropanoid genes were not affected in transgenic plants

qRT-PCR was conducted to examine the transcriptional response, if any, of representative phenylpropanoid genes in transgenic plants with increased hydroxycinnamoyl-glucose esters. These included two isoforms each of the phenylalanine ammonia-lyase (PAL), 4-coumarate:CoA ligase (4CL), and caffeoyl-CoA 3-*O*-methyltransferase families. N-sensitive expression responses were observed for *PAL* and *4CL* in an isoform- and tissue-dependent manner, but no transgenic effects were detected for any of the phenylpropanoid genes tested (Supplementary Fig. S8). The results suggested that elevated hydroxycinnamoyl-glucose accumulation was driven by *PfaGT1-316* overexpression and redirection of phenylpropanoid pathway intermediates, without stimulating phenylpropanoid biosynthesis at the transcriptional level.

## Discussion

### PfaGT1-316 encodes a hydroxycinnamate glycosyltransferase

Phylogenetic, biochemical, and transgenic analyses provided strong support that *PfaGT1-316* encodes a hydroxycinnamate glycosyltransferase. The ability of PfaGT1-316 to accept multiple hydroxycinnamate/benzoate substrates *in vitro* was corroborated *in vivo*, as *PfaGT1-316* overexpression in transgenic poplars increased accumulation of caffeoyl-glucose, 4-coumaroyl-glucose, cinnamoyl-glucose, and several other less abundant hydroxycinnamoyl/benzoyl-glucose esters. A similar multisubstrate utilization pattern has been noted for several UGT84A orthologues (Lim *et al.*, 2001; Lunkenbein *et al.*, 2006; Mittasch *et al.*, 2007), consistent with the propensity of many GT1 members to exhibit regioselectivity rather than true specificity (Vogt and Jones, 2000; Lim *et al.*, 2003). However, this differs from the *Arabidopsis* UGT84A2 and oilseed rape UGT84A9 that exhibit a much more restricted substrate preference for sinapic acid (Lim *et al.*, 2001; Mittasch *et al.*, 2007). The *Populus GT1-315/316* transcripts were detected in a wide range of tissues, especially leaves, which contrasts with the seed-, seedling-, and/or flower-preferential expression of *Arabidopsis UGT84A2* (Schmid *et al.*, 2005), oilseed rape *UGT84A10* (Mittasch *et al.*, 2007), and strawberry UGT84A6 (Lunkenbein *et al.*, 2006). Thus, while the UGT84A family appears evolutionarily conserved (Fig. 2), variation in tissue expression and substrate utilization preference exists among isoforms and may confer species- or tissue-specific roles. The broad expression and substrate utilization patterns of PfaGT1-316 suggest that it may play a more general role of modulating phenylpropanoid metabolism.

### *PfaGT1-316* overexpression affected phenylpropanoid metabolism

Overexpression of *PfaGT1-316* resulted in increases of hydroxycinnamoyl- and benzoyl-glucose esters in all *Populus* tissues examined, regardless of N status. Levels of various phenylpropanoid derivatives and conjugates were also increased. However, rutin and kaempferol-3-*O*-rutinoside, the two most abundant flavonoid glycosides in leaves, decreased. As expression of phenylpropanoid pathway genes was unaffected in transgenic plants, the metabolic effects observed appear to be direct consequences of elevated PfaGT1-316 glycosylation activity. The findings that hydroxycinnamoyl-glucose esters were increased at the expense of the abundant flavonoid rutinosides are consistent with both metabolite pools being dependent on aglycone hydroxycinnamates and UDP-glucose for their synthesis. Reduced flavonoid rutinoside accumulation likely led to secondary trade offs within the flavonoids, resulting in increased accrual of many other less abundant flavonoid conjugates. Metabolic trade offs between distinct phenylpropanoid pools have been frequently reported (Xie *et al.*, 2003; Tattini *et al.*, 2004; Clauß *et al.*, 2011; Kosonen *et al.*, 2012) and, in several cases, the trade offs have been associated with altered phenylpropanoid glycosylation (Sinlapadech *et al.*, 2007; Griesser *et al.*, 2008; Lanot *et al.*, 2008; Payyavula *et al.*, 2009). For instance, mutation of *UGT84A2* in *Arabidopsis* resulted in reduced sinapoyl-glucose and its malate and choline esters, while an unusual flavonoid, sinapic acid-derived polyketide, hyperaccumulated in the trichomes (Sinlapadech *et al.*, 2007). Together, these findings are in line with the highly plastic nature of the phenylpropanoid network in response to genetic or environmental perturbations (Vogt, 2010) and suggest that glycosylation of small phenolics can modulate a multitude of cellular and metabolic responses to affect phenylpropanoid pool composition.

### Hydroxycinnamate glycosylation as a mediator of stress response

The initial identification of *GT1-316* from poplar stress transcriptomes (Supplementary Fig. S1 available at *JXB* online), its N-sensitive expression in multiple genotypes (Figs 1 and 4A), and the large overlap of metabolic response between N-stressed and *GT1-316*-overexpressing poplars (Fig. 5F) provide multiple lines of evidence to support a role of GT1-316 in stress response. *Populus*, more than many other species, depends on large, constitutive, yet dynamic phenylpropanoid pools for stress response (reviewed in Tsai *et al.*, 2006; Douglas *et al.*, 2011). Given the central position of free hydroxycinnamates that support multiple phenylpropanoid branchways, GT1-316 activity can potentially modulate stress-induced shifts in carbon partitioning through its action on aglycone hydroxycinnamate pools. This is consistent with elevated expression of several *UGT84A* genes in response to UV-B or a pharmacologically induced oxidative burst (Lunkenbein *et al.*, 2006; Meißner *et al.*, 2008) and with the roles of hydroxycinnamoyl-glucoses as UV protectants (Landry *et al.*, 1995; Lehfeldt *et al.*, 2000; Meißner *et al.*, 2008) and radical scavengers (Braham *et al.*, 2005; Kylli *et al.*, 2008; D’Abrosca *et al.*, 2010). Recent studies showed that hydroxycinnamoyl-glucose esters serve as acyl donors in anthocyanin acylation, as *Arabidopsis* mutant and transgenic oilseed rape defective in *UGT84A2* and *UGT84A9*, respectively, exhibited reduced accrual of not only sinapoyl-esters, but also sinapoylated anthocyanins (Wolfram *et al.*, 2010; Yonekura-Sakakibara *et al.*, 2012). Acylation with hydroxycinnamates is a common modification of phenylpropanoids (D’Auria, 2006; Tsai *et al.*, 2006), known to alter the bioactivity, stability, and/or absorbance of the acceptor substrates (reviewed in Yoshida *et al.*, 2009). Aromatic acylation of phenylpropanoids depends on either hydroxycinnamoyl-CoA or hydroxycinnamoyl-glucose esters as the high-energy acyl donors (Teusch *et al.*, 1987; Gläßgen and Seitz, 1992; Mock and Strack, 1993). This places UGT84A in a position to modulate the availability of hydroxycinnamoyl donors for phenylpropanoid acylation. Thus, GT1-316/UGT84A17 could play both direct and indirect roles in modulating phenylpropanoid synthesis, modification, bioactivity, and/or stability in response to stress and glucose availability cues.

In summary, overexpression of GT1-316 caused changes in phenylpropanoid composition in *Populus*, suggesting an important role of glycosylation in phenylpropanoid metabolism. *Populus* GT1-316, like its UGT84A orthologues, is developmentally and environmentally regulated. Given the propensity of phenylpropanoids to exhibit taxon-specific diversity (Tsai *et al.*, 2006), the current results suggest that the UGT84A subfamily, while evolutionarily conserved, may serve species-specific functions in modulating phenylpropanoid metabolism in response to developmental and environmental cues. The work presented here opens up new prospects to explore the physiological roles of diverse hydroxycinnamate derivatives in stress responses of the GT1-316-overexpressing *Populus*.

## Supplementary material

Supplementary data are available at *JXB* online.

Supplementary Table S1. Primer information.

Supplementary Table S2. Characteristics of PfaGT1-316 glycosylation products confirmed by HPLC-MS/TOF.

Supplementary Table S3. List of LC-MS/TOF-identified metabolites significantly changed in transgenic plants.

Supplementary Fig. S1. Expression response of two GT1-315/316 probe-sets to various stress treatments in multiple *Populus* genotypes.

Supplementary Fig. S2. HPLC-MS/TOF confirmation of PfaGT1-316 enzyme assay products.

Supplementary Fig. S3. Alkaline hydrolysis of PfaGT1-316 assay products to confirm glucose-ester linkage.

Supplementary Fig. S4. Screening of independent GT1-316 transgenic lines.

Supplementary Fig. S5. Additional growth data.

Supplementary Fig. S6. Analysis of wall-bound phenolics in xylem of WT and transgenic *Populus* grown under different N regimes.

Supplementary Fig. S7. Effects of *PfaGT1-316* overexpression on major phenylpropanoid products.

Supplementary Fig. S8. Relative transcript abundance of representative phenylpropanoid pathway genes in WT and transgenic *Populus* grown under different N regimes.

Supplementary Data
