# Supplementary material for: Stress-responsive hydroxycinnamate glycosyltransferase modulates phenylpropanoid metabolism in Populus
Source: J Exp Bot. 2014 May 6;65(15):4191–200. doi: 10.1093/jxb/eru192 (PMC4112628; doi:10.1093/jxb/eru192)
Supplement: Supplementary Data [file supp_65_15_4191__index.html]

Stress-responsive hydroxycinnamate glycosyltransferase modulates phenylpropanoid metabolism in Populus — Stress-responsive hydroxycinnamate glycosyltransferase modulates phenylpropanoid metabolism in Populus — Supplementary Data 

# Stress-responsive hydroxycinnamate glycosyltransferase modulates phenylpropanoid metabolism in *Populus*

## Supplementary Data

Data files

**Files in this Data Supplement:**

- Supplementary Data - Supplementary Data
- Supplementary Data - Supplementary Data
