# Supplementary material for: Stress-responsive hydroxycinnamate glycosyltransferase modulates phenylpropanoid metabolism in Populus
Source: J Exp Bot. 2014 May 6;65(15):4191–200. doi: 10.1093/jxb/eru192 (PMC4112628; doi:10.1093/jxb/eru192)
Supplement: Supplementary Data [file supp_eru192_jexbot112201_file001.pdf]

**Table S1.** Primer information

| Gene | Forward and reverse primers | Purpose |
|---|---|---|
| GT1-316 | F: <u>GGATCC</u>CTGGTTTCCGAGTCTCTTGG<br>R: <u>CCCGGG</u>TCATGCCGATGACCCCAC | recombinant protein expression |
| GT1-316 | F: ATGGTTTCCGAGTCTCTTGG<br>R: TCATGCCGATGACCCCAC | binary vector construction |
| GT1-315/316 cluster | F: CCTAAGGCAGTGGAAATGAAGSAG<br>R: CCAGTTCADCSACTTYCTCRHTGG | qRT-PCR |
| PAL1 | F: ATGTCTTTGCTTACGCCGATGACC<br>R: TCATATGCTGCTCTTGCGCTCTCA | qRT-PCR |
| PAL2 | F: AGAGAGTCCTGACAATGGGCTTCA<br>R: GYTGGGTTGCCRTTCTCAAKTTCA | qRT-PCR |
| 4CL1 | F: TAGTGAAATCAGAAAAGTCTCAGG<br>R: CGCAAGTATTAAAGAAATAAGACAG | qRT-PCR |
| 4CL2 | F: TATTCCCAAATCGGCTTCTGG<br>R: GGCAAGCTTGGCTCTCAGGTC | qRT-PCR |
| CCoAOMT1 | F: RCCRATGAGGAAGTATGTGAGGTA<br>R: CAGATAAYAATAYTGGCAGGTCCC | qRT-PCR |
| CCoAOMT2 | F: RCCRATGAGGAAGTATGTGAGGTA<br>R: TATAATATHGGCAGRGAATGCAGCCC | qRT-PCR |
| elongation factor 1-β | F: GACCTKGTATCAGTGGATTCCCTC<br>R: GAACAGAGGCACAAGATTACCAGG | qRT-PCR |
| α-tubulin 4 | F: CTTGCAATAGMATTCATCTGGGAAATGGT<br>R: CTTCAAATACGAAGACAGACATAGTCTTG | qRT-PCR |
| ubiquitin-conjugating enzyme E2 | F: CTGAAGAAGGAGATGACARCMCCA<br>R: GCATCCCTTCAACACAGTTTCAMG | qRT-PCR |

**Supplemental Table 2.** Characteristics of PfaGT1-316 glycosylation products confirmed by HPLC-MS/TOF.

| Product name (substrate) | RT (min) | detected *m/z* | ion | expected *m/z* | Δ mass (ppm) | Product UV/Vis max | Substrate UV/Vis max | Molecular formula |
|---|---|---|---|---|---|---|---|---|
| caffeoyl-glucose (caffeic acid) | 2.50 | 341.0865 | $[M-H]^-$ | 341.0873 | -2.35 | 335, 212, 300 | 323, 218, 294-ish, 235-240-ish | C15H18O9 |
| 2-coumaroyl-glucose (2-coumaric acid) | 3.94 | 325.0934 | $[M-H]^-$ | 325.0923 | 3.38 | 281, 330, 229, 218-ish | 276, 324, 216 | C15H18O8 |
| 4-coumaroyl-glucose (4-coumaric acid) | 3.11 | 325.0929 | $[M-H]^-$ | 325.0923 | 1.85 | 315, 228, 296-ish, 210-ish | 310, 225, 290ish, 212ish | C15H18O8 |
| 4-hydroxybenzoyl-glucose (4-hydroxybenzoic acid) | 1.73 | 299.0764 | $[M-H]^-$ | 299.0767 | -1.00 | 260, 210 | 256, 210ish | C13H16O8 |
| sinapoyl-glucose (sinapic acid) | 3.47 | 385.1126 | $[M-H]^-$ | 385.1135 | -2.34 | 331, 225, 240-ish | 324, 225, 235-ish | C17H22O10 |
| feruloyl-glucose (ferulic acid) | 3.42 | 355.1042 | $[M-H]^-$ | 355.1029 | 3.66 | 330, 220, 295-ish, 240-ish | 322, 219, 295-ish, 232-ish | C16H20O9 |
| cinnamoyl-glucose (cinnamic acid) | 4.95 | 355.1043 | $[M+CHOOH-H]^-$ | 355.1029 | 3.94 | 282 | 277, 218 | C15H18O7 |
| benzoyl-glucose (benzoic acid) | 3.45 | 329.0888 | $[M+CHOOH-H]^-$ | 329.0873 | 4.56 | 232, 276 | 228, 273 | C13H16O7 |
| naringenin glucoside* (naringenin) | 5.58 | 433.1134 | $[M-H]^-$ | 433.1135 | -0.23 | 284, 223, 332-ish | 288, 222, 327-ish | C21H22O10 |
| 4-hydroxybenzaldehyde glucoside* (4-hydroxybenzaldehyde) | 1.79 | 329.0890 | $[M+CHOOH-H]^-$ | 329.0873 | 5.17 | 265, 213, 220-ish, 280-ish | 283, 220, 200, 268-ish | C13H16O7 |
| kaempferol glucoside* (kaempferol) | 5.54 | 447.0951 | $[M-H]^-$ | 447.0927 | 5.37 | 364, 263, 249-ish, 315-ish | 363, 265, 320-ish, 300-ish | C21H20O11 |

* The exact identity (the natural of the glycosidic linkage) of these metabolites was not verified.

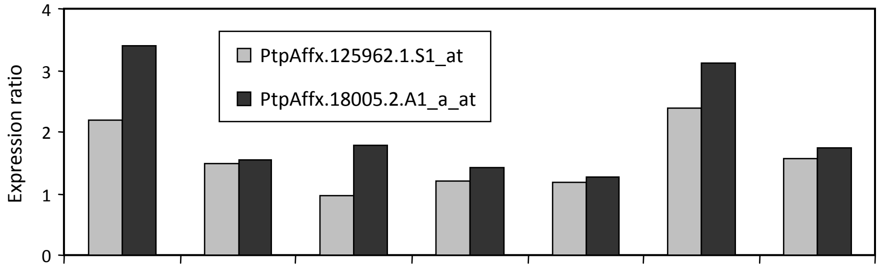


| Tissue | YL | EL | YL | EL | YL | EL | EL |
|---|---|---|---|---|---|---|---|
| Stress | 1-wk N stress | | 1-wk N stress | | 1-wk post wounding | | 90-hr post detopping |
| Genotype | *P. fremontii x angustifolia* clone 1979 | | *P. fremontii x angustifolia* clone 3200 | | *P. fremontii x angustifolia* clone RM5 | | *P. tremuloides* clone 271 |

**Fig. S1.** Expression response of two *GT1-315/316* probe-sets to various stress treatments in multiple *Populus* genotypes. Expression ratio of stressed-to-unstressed samples was calculated using MAS5-processed Affymetrix microarrays, based on the mean of two biological replicates. YL, young leaves; EL, expanding leaves.

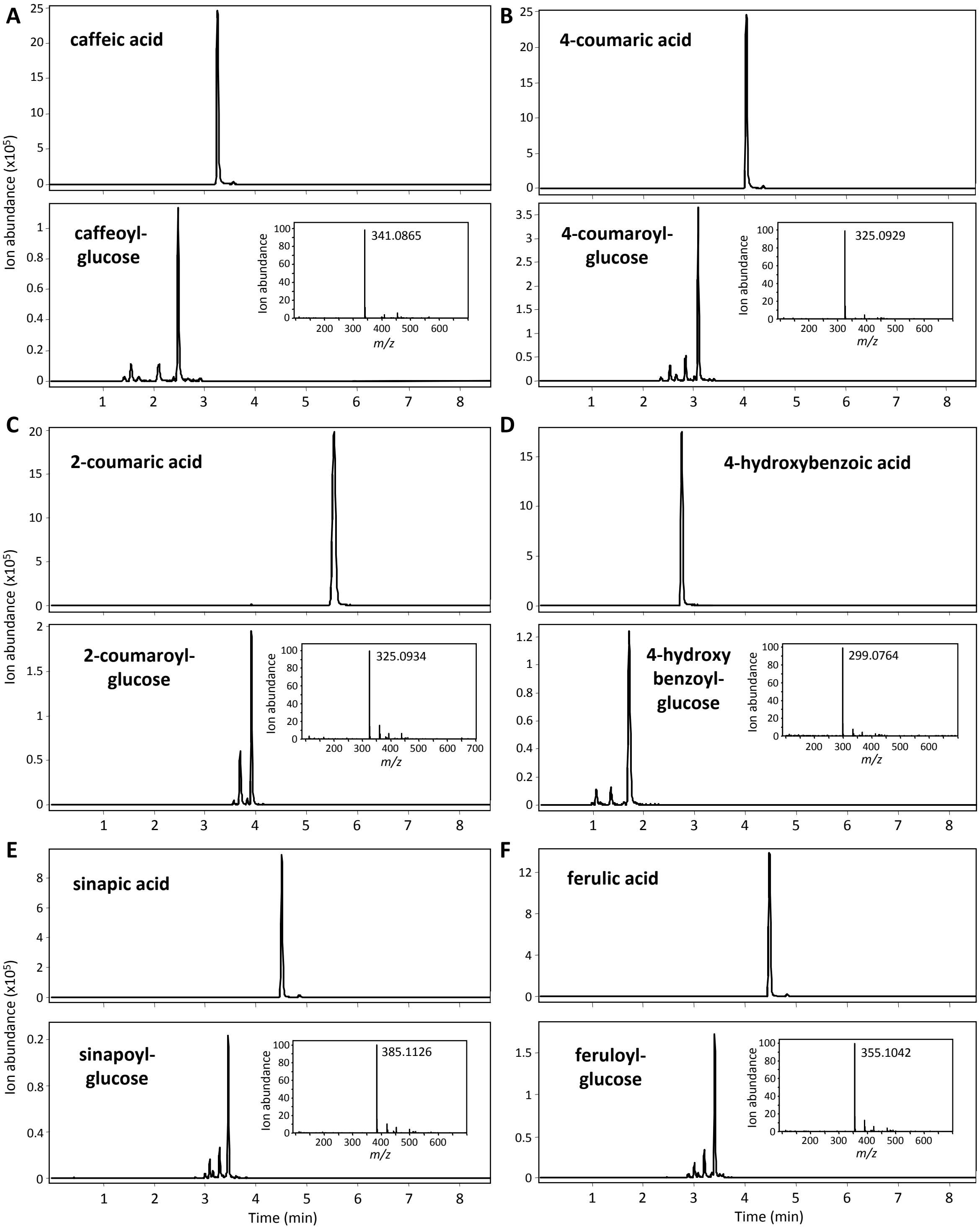


**Fig. S2.** HPLC-MS/TOF confirmation of PfaGT1-316 enzyme assay products. Extracted ion chromatograms for the substrate aglycone (top), glycosylation product (bottom) and mass spectra of the product (inset) are shown for (A) caffeic acid, (B) 4-coumaric acid, (C) 2-coumaric acid, (D) 4-hydroxybenzoic acid, (E) sinapic acid, (F) ferulic acid, (G) cinnamic acid, (H) benzoic acid, (I) naringenin, (J) 4-hydroxybenzaldehyde, (K) kaempferol. For each substrate there was one major product peak.

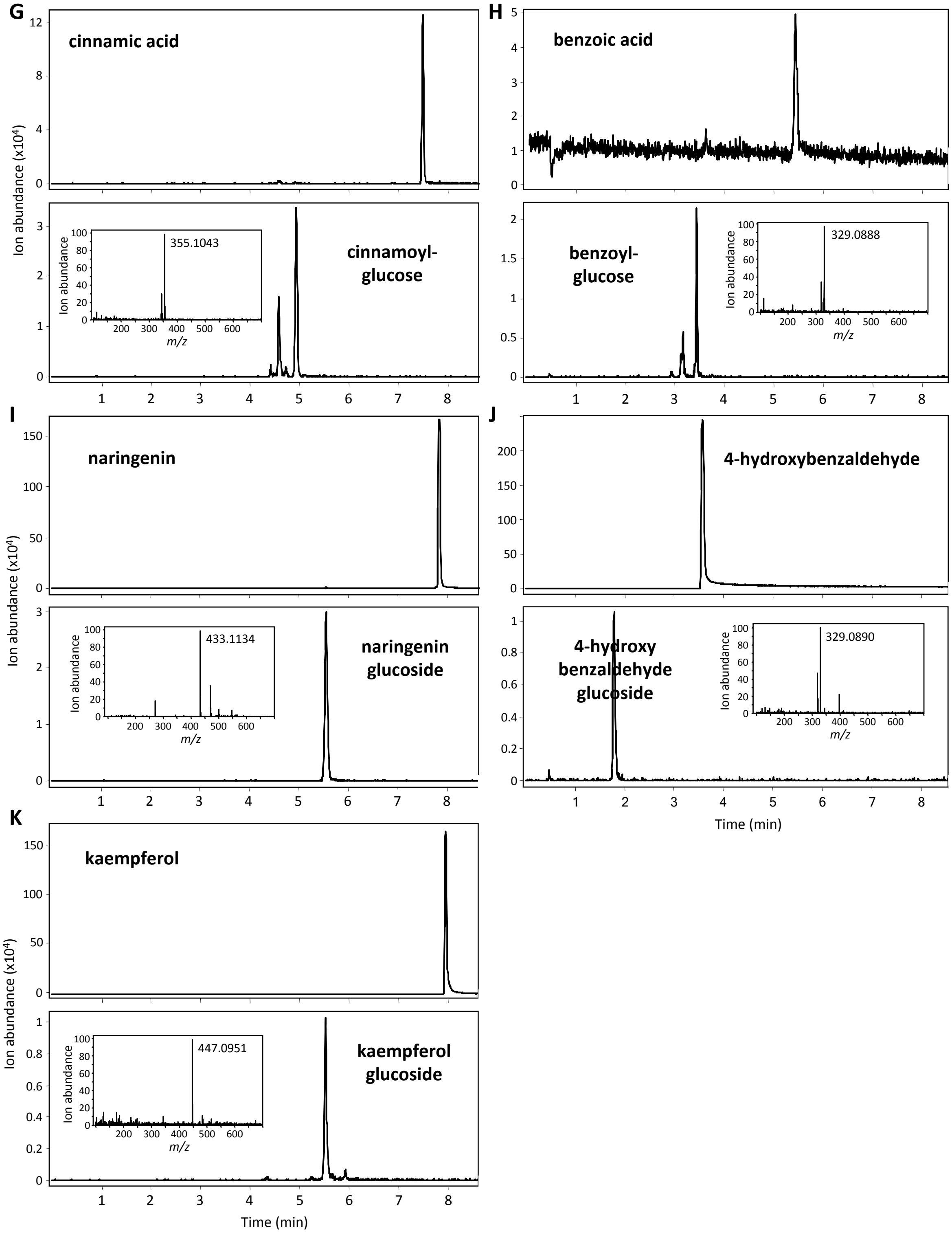


**Fig. S2.** (continued).

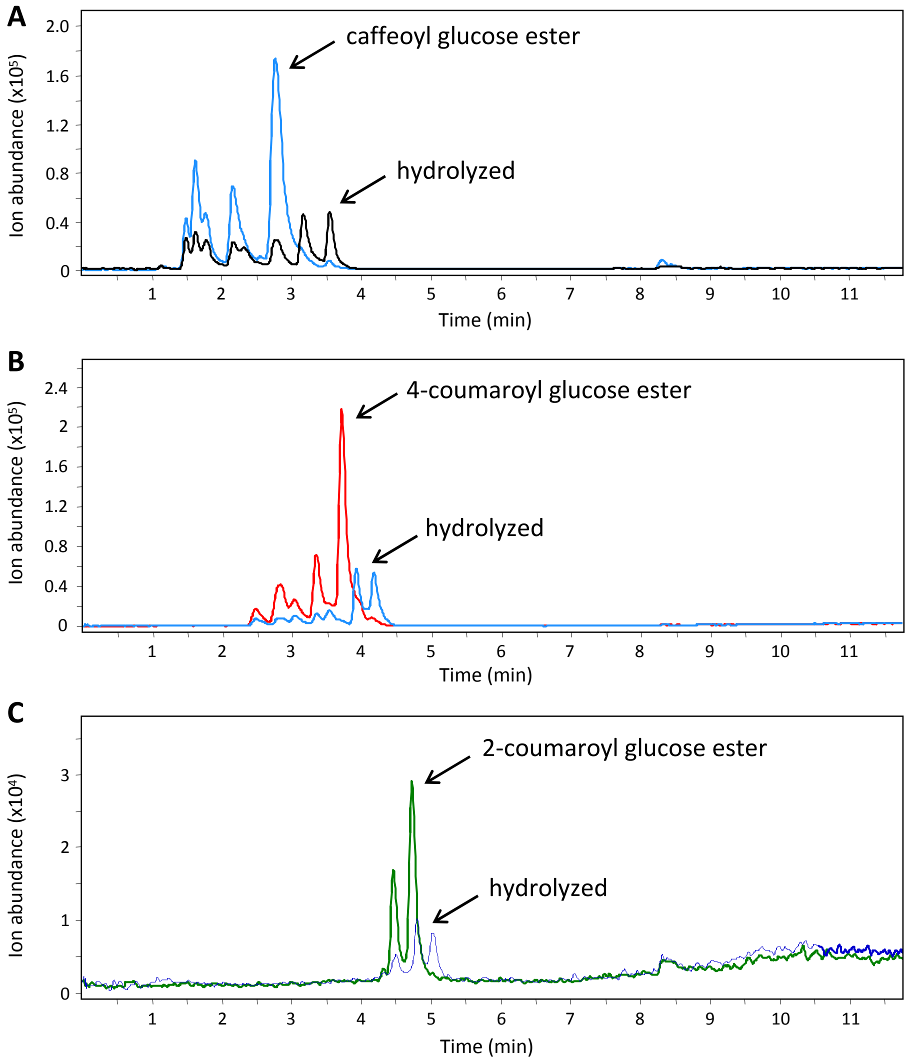


**Fig. S3.** Alkaline hydrolysis of PfaGT1-316 assay products to confirm glucose-ester linkage. Extracted ion chromatograms are shown for the major ion of glycosylation products of (A) caffeic acid, (B) 4-coumaric acid, and (C) 2-coumaric acid, with NaOH-hydrolyzed product chromatograms overlayed. Since the NaOH treatment cleaves only glucose ester, but not *O*-glucoside bonds, the digestibility of the PfaGT1-316 products strongly suggests that PfaGT1-316 catalyzes the formation of phenolic glucose esters, rather than phenolic *O*-glucosides.

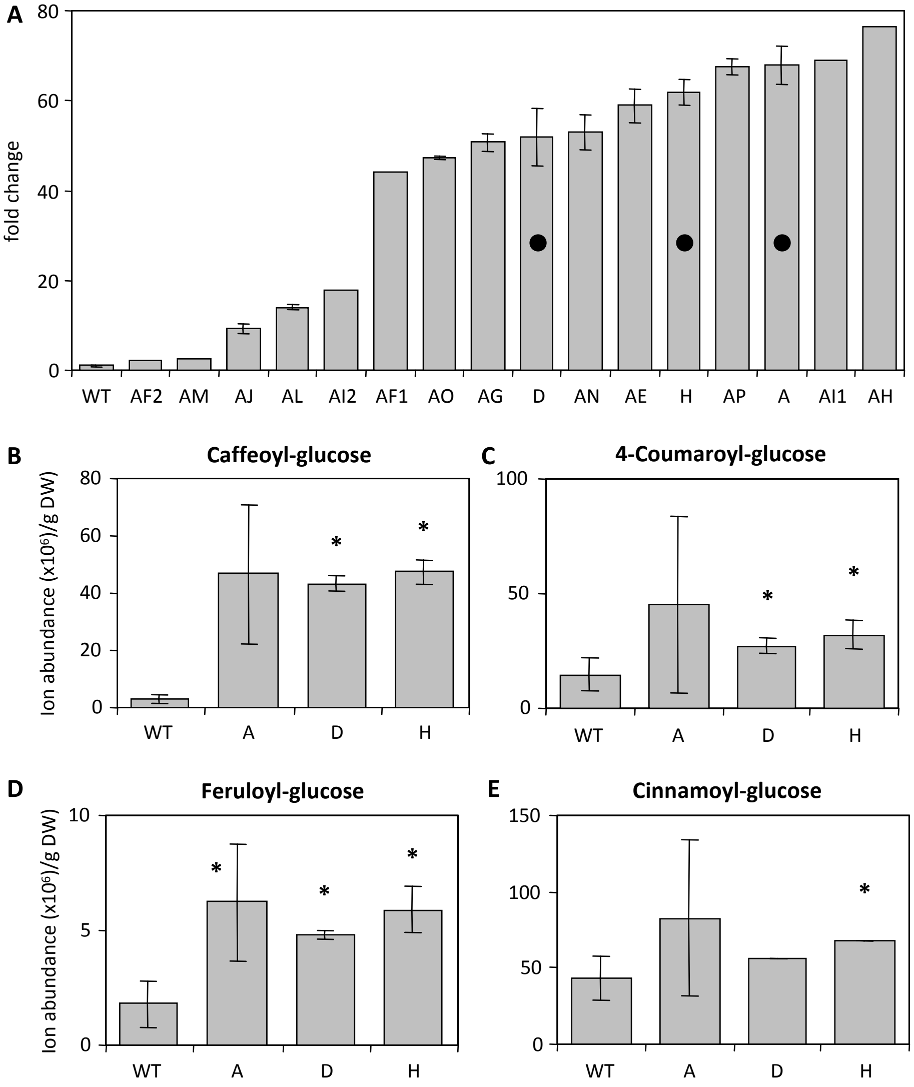


**Fig. S4.** Screening of independent GT1-316 transgenic lines. (A) qRT-PCR analysis of *GT1-315/316* transcript abundance in leaves (LPI-5) of WT and 16 independent primary transformants (n = 2 where error bars are shown). Lines marked with dots were selected for further analysis. (B) to (E), HPLC-MS/TOF analysis of caffeoyl- (B), 4-coumaroyl- (C), feruloyl- (D) and cinnamoyl-glucose esters (E) in WT and three transgenic lines. Statistical significance ($P < 0.05$) is marked by asterisks.

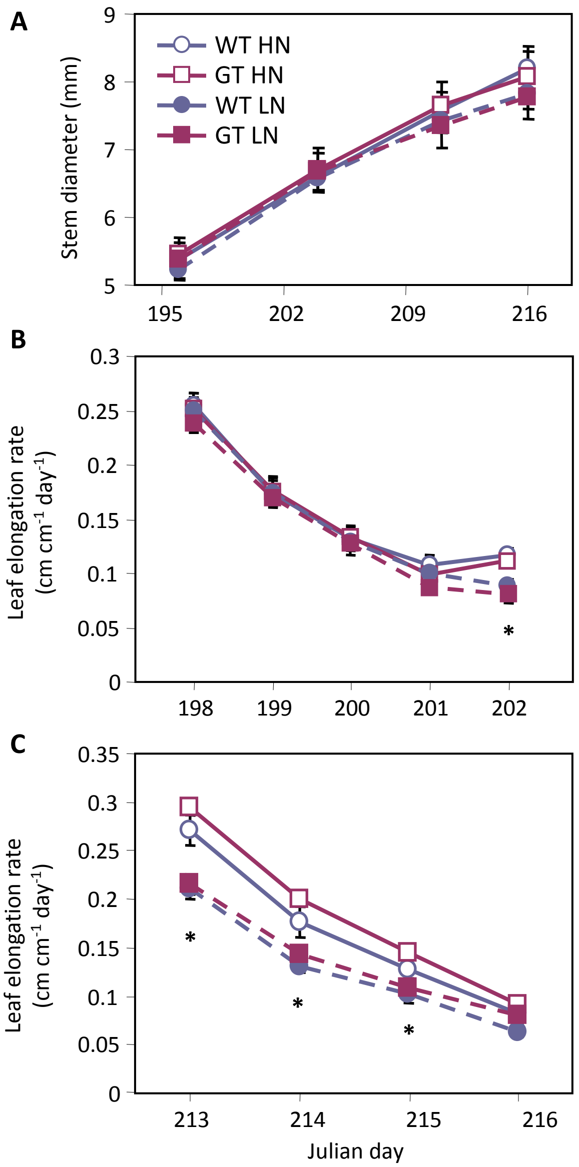


**Fig. S5.** Additional growth data. (A) Stem diameter and (B)-(C) leaf elongation rate at the onset (B) and after two weeks (C) of low-N treatments in WT and PfaGT1-316 over-expressing transgenic (GT) plants. Data represent means and standard errors of n= 15 in (A) and n= 5-6 in (B) and (C). N treatment effects are indicated by asterisks ($P < 0.05$).

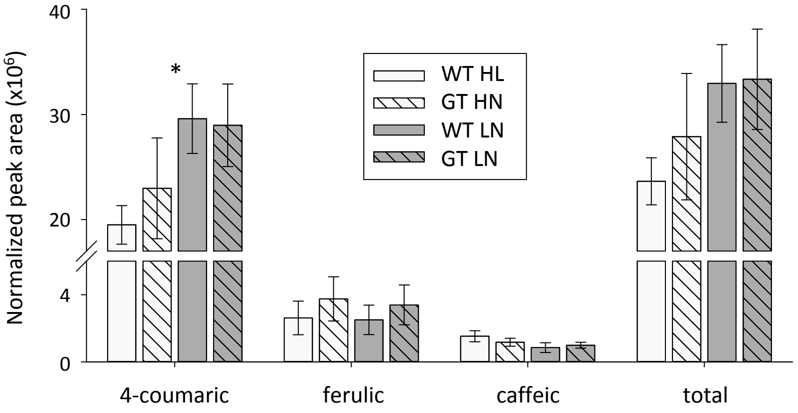


**Fig. S6.** Analysis of wall-bound phenolics in xylem of WT and transgenic *Populus* grown under different N regimes. There was no statistically significant transgenic effect. N treatment effects were indicated by an asterisk ($P < 0.05$).

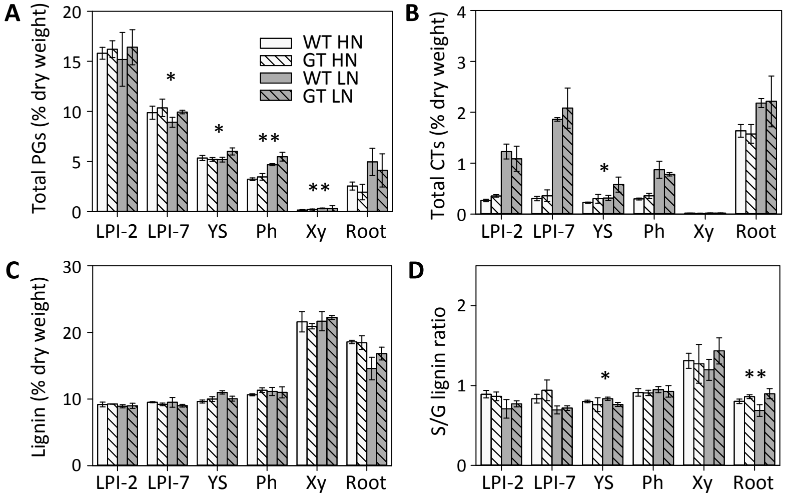


**Fig. S7.** Effects of PfaGT1-316 over-expression on major phenylpropanoid products. Shown are (A) phenolic glycoside (PG), (B), condensed tannin (CT), and (C) lignin content, and (D) S/G lignin ratio. Bars indicate means ± SE (n= 3). Asterisks indicate significant genotypic differences from two way ANOVA (genotype × N treatment); * $P < 0.1$; ** $P < 0.05$.

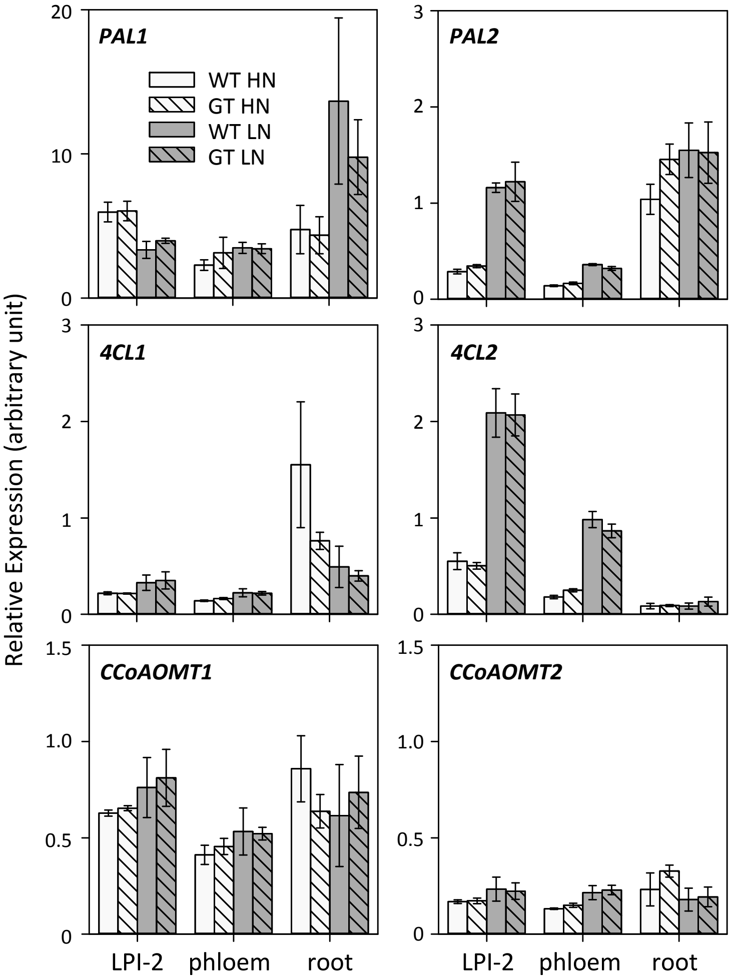


**Fig. S8.** Relative transcript abundance of representative phenylpropanoid pathway genes in WT and transgenic *Populus* grown under different N regimes. Two isoforms each for PAL (phenylalanine ammonia lyase), 4CL (4-coumarate:CoA ligase) and CCoAOMT (caffeoyl-CoA 3-*O*-methyltransferase) were analyzed. Numbering of isoforms is based on Tsai *et al*. (2006).

**Tsai CJ, Harding SA, Tschaplinski TJ, Lindroth RL, Yuan Y**. 2006. Genome-wide analysis of the structural genes regulating defense phenylpropanoid metabolism in *Populus*. New Phytologist 172, 47-62.
